# A SYSTEMATIC REVIEW OF THE LITERATURE ON ACADEMIC RETIREMENT

**DOI:** 10.1093/geroni/igae098.2514

**Published:** 2024-12-31

**Authors:** Michelle Silver

**Affiliations:** University of Toronto, Toronto, Ontario, Canada

## Abstract

**Objectives:**

The number of academics reaching traditional retirement age has been increasing thus motivating the need to better understand retirement experiences among higher education faculty. This review paper aims to: (i) identify the types of studies and traits being measured in studies of academic retirement, (ii) understand the determinants of retirement, including pathways and obstacles, among academics being measured in the literature, (iii) map the most prevalent types of studies and findings regarding academic retirement, and (iv) appraise the current state of the literature.

**Methods:**

We conducted a systematic review of the literature to address our research objectives. Studies were identified from multiple database using search terms regarding retirement among academic faculty. Included studies examined determinants of academic retirement, perspectives, and/or retirement experiences among academics. Four reviewers screened the studies for inclusion and completed the data extraction.

**Results:**

A total of 102 papers met the inclusion criteria. Most studies were based on cross-sectional survey data, followed by qualitative interviews; quantitative, qualitative, and mixed-methods research were included. Commonly measured determinants included age and spousal retirement timing. A mapping of the literature indicates a focus on geographical and/or subfield areas. Conclusions: Insights regarding academic’s retirement experiences provide insights about varying retirement pathways and can help elucidate retirement pathways that enhance transitions particularly for those in professions with generally high autonomy. Future research ought to focus on supports to help retain mature workers within academia and overall determinants of satisfaction with retirement among academics.

